# A MEMS Electrochemical Angular Accelerometer Leveraging Silicon-Based Three-Electrode Structure

**DOI:** 10.3390/mi13020186

**Published:** 2022-01-26

**Authors:** Mingwei Chen, Anxiang Zhong, Yulan Lu, Jian Chen, Deyong Chen, Junbo Wang

**Affiliations:** 1 State Key Laboratory of Transducer Technology, Aerospace Information Research Institute, Chinese Academy of Sciences, Beijing 100190, China; chenmingwei19@mails.ucas.ac.cn (M.C.); zhonganxiang19@mails.ucas.ac.cn (A.Z.); luyl@aircas.ac.cn (Y.L.); chenjian@mail.ie.ac.cn (J.C.); 2 School of Electronic, Electrical and Communication Engineering, University of Chinese Academy of Sciences, Beijing 100049, China

**Keywords:** angular accelerometer, electrochemical principle, MEMS, silicon based three-electrode structure, high performance

## Abstract

This paper developed an electrochemical angular micro-accelerometer using a silicon-based three-electrode structure as a sensitive unit. Angular acceleration was translated to ion changes around sensitive microelectrodes, and the adoption of the silicon-based three-electrode structure increased the electrode area and the sensitivity of the device. Finite element simulation was conducted for geometry optimization where the anode length, the orifice diameter, and the orifice spacing of the sensitive unit were determined as 200 μm, 80 μm, and 500 μm, respectively. Microfabrication was conducted to manufacture the silicon-based three-electrode structure, which then was assembled to form the electrochemical angular micro-accelerometer, leveraging mechanical compression. Device characterization was conducted, where the sensitivity, bandwidth, and noise level were quantified as 290.193 V/(rad/s^2^) at 1 Hz, 0.01–2 Hz, and 1.78 × 10^−8^ (rad/s^2^)/Hz^1/2^ at 1 Hz, respectively. Due to the inclusion of the silicon-based three-electrode structure, compared with previously reported electrochemical angular accelerometers, the angular accelerometer developed in this article was featured with a higher sensitivity and a lower self-noise level. Therefore, it could be used for the measurement of low-frequency seismic rotation signals and played a role in the seismic design of building structures.

## 1. Introduction

The measurements of seismic vibrations are of great significance for the studies of seismology and the seismic designs of engineering structures. The current ground motion detection is mainly based on the translational component [1], ignoring the rotational component (a key component of seismic vibration), and thus, the impacts of rotational vibrations are rarely considered in the seismic design of building structures, resulting in severe building damages in histories of seismic vibrations [2]. Therefore, in addition to studying the seismic translational components, the measurements of the rotation component are also of great significance [3].

Angular accelerometers are currently used seismometers that measure the rotation components of seismic vibrations, which are divided into angular accelerometers based on solid and liquid inertial masses [4]. Angular accelerometers based on solid inertial masses had poor impact resistance, large volumes, and low sensitivities at the low-frequency domain [5,6,7,8,9]. Differently, the inclusion of liquid inertial masses into angular accelerometers can lead to increases in both impact resistance and sensitivities at the low-frequency domain due to electrochemical principles [10,11,12,13]. First, angular accelerometers using liquid inertial mass have strong shock resistance due to there being no mechanical structure [13]. Secondly, the relationship between the concentration gradient of the electrochemical angular accelerometers and the generated current is based on Faraday’s law, so a small concentration change can generate a large current: that is, the electrochemical angular accelerometers have high sensitivities [14].

Conventionally, sensitive electrodes made of ceramic sintering were used in angular accelerometers based on liquid masses, which demonstrated a high sensitivity at the low-frequency domain and at the same time suffered from key limitations of structure opination due to complex fabrication processes [15,16]. In order to address this issue, microfabrication was used to manufacture sensitive electrodes which were integrated into angular accelerometers with fine-tuned structures [17,18]. However, these previously developed angular accelerometers with microfabricated microelectrodes suffered from electrode areas and compromised device sensitivities because of the planar setup of sensitive microelectrodes.

Therefore, this paper presented an electrochemical angular micro-accelerometer with a bulk setup of sensitive microelectrodes. More specifically, the electrode-based sensitive unit was an integrated porous structure of bulk silicon where two cathodes were positioned on the front and back sides and one anode was positioned on the side wall of the orifice. Compared to planar microelectrodes, the sensitive unit adopted a bulk silicon structure to increase the effective electrode area, thereby increasing the sensitivity. In comparison to the counterpart based on ceramic sintering with complex fabrication, the sensitive electrodes in this study can be made by standard microfabrication processes. Compared with the above two angular accelerometers, the sensitivity of the angular accelerometer in this paper was improved by more than an order of magnitude, so the low-frequency seismic rotation signal could be detected by it more sensitively.

## 2. Working Principle

**Figure 1:**
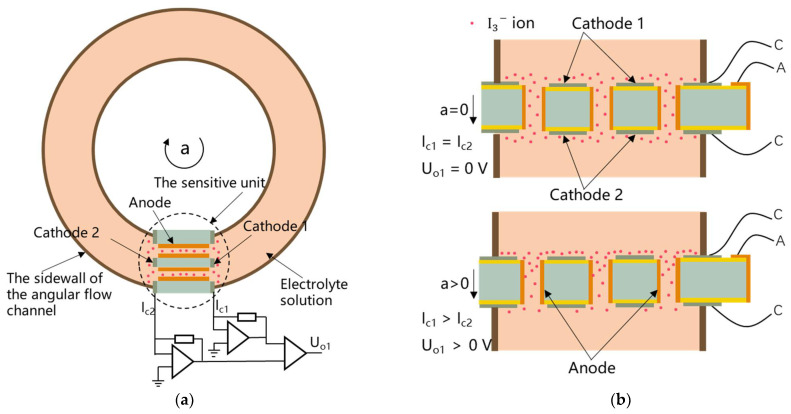
(**a**) Working principle of the MEMS electrochemical angular accelerometer, mainly composed of a silicon-based three-electrode structure fixed in an angular flow channel, filled with an electrolyte solution. (**b**) In operation, an incoming angular vibration causes an accumulation of $I_{3}{}^{-}$ ions on one cathode and a depletion of $I_{3}{}^{-}$ ions on the other cathode. Since $I_{3}{}^{-}$ ions are oxidized into ${\;I}^{-}$ ions on cathodes, imbalanced $I_{3}{}^{-}$ ions on two cathodes, two cathodes produce a differential current output proportional to the amplitude of the angular vibration.

The electrochemical angular micro-accelerometer with the bulk setup of sensitive microelectrodes is shown in Figure 1a. The micro-accelerometer is mainly composed of an electrode-based sensitive unit fixed in an angular flow channel that is filled with an electrolyte solution of ${\;I}^{-}$ and $I_{3}{}^{-}$. The electrolyte is a mixed solution of $I_{2}$ and KI. Since the concentration of KI is much higher than that of $I_{2}$, $I_{2}$ in the electrolyte exists in the form of $I_{3}{}^{-}$.

Figure 1b shows a cross-sectional view of the sensitive unit, which is obtained by rotating the sensitive unit in the dotted circle in Figure 1a by 90 degrees counterclockwise. Among them, the tan color represents the sidewall of the angular flow channel. The sensitive unit was based on a silicon layer with multiple through-holes termed as “orifice”. The front and back sides of the silicon wafer were covered with an insulation layer, which is followed by the deposition of platinum as two cathodes. In addition, the side walls of orifices were deposited with platinum to form one anode. In the end, electrical routing of the anode was realized by the bulk silicon layer, while two cathodes were directly connected with surrounding pins, forming the sensitive unit with the cathode–anode–cathode set up.

Figure 1b also shows the working principle of an electrochemical angular micro-accelerometer based on well-established electrochemical principles [13]. Briefly, when there is an angular vibration, there is an accumulation of $I_{3}{}^{-}$ ions on one cathode and a depletion of $I_{3}{}^{-}$ ions on the other cathode. Since $I_{3}{}^{-}$ ions are reduced into ${\;I}^{-}$ ions on cathodes, imbalanced $I_{3}{}^{-}$ ions on two cathodes leads to imbalanced current output of two cathodes. The currents of two cathodes $I_{c1}$ and $I_{c2}$ are converted into a differential voltage output $U_{o1}$ proportional to the input angle vibration signal through a current–voltage conversion circuit and a differential amplifier circuit (see Figure 1a).

In this electrochemical angular micro-accelerometer with the bulk setup of sensitive microelectrodes, there are three key geometrical parameters of anode length ($L_{a}$), orifice diameter ($d_{m}$), and orifice spacing ($L_{s}$). Since these parameters can affect the performances (e.g., sensitivity and bandwidth) of the angular micro-accelerometer in a comprehensive manner, numerical simulations were conducted in the following section for geometrical optimization.

## 3. Numerical Simulation

The software used for the simulation is COMSOL finite element analysis software. The electrochemical angular micro-accelerometer with the bulk setup of sensitive microelectrodes was divided into a mechanical module and an electromechanical module, and finite element analysis based on physical fields of rotating machinery and laminar flow as well as the physics fields of tertiary current distribution and laminar flow were conducted for simulation.

**Figure 2:**
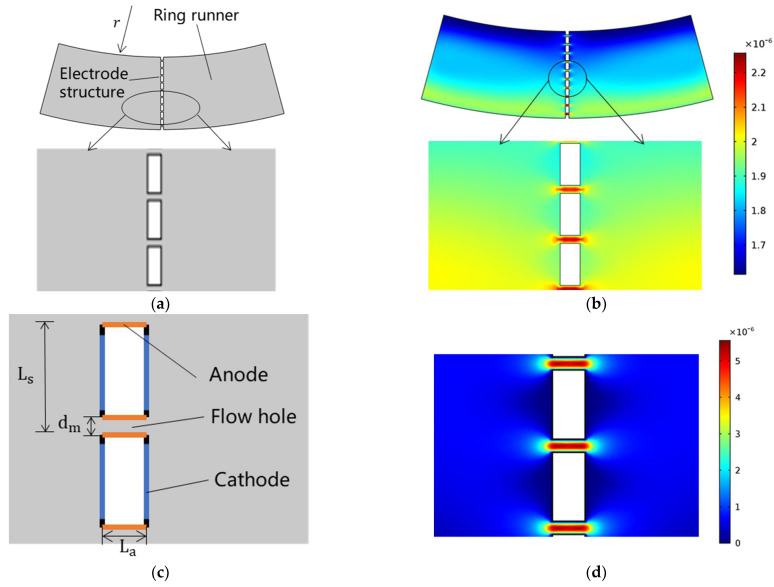
Numerical simulation of the MEMS electrochemical angular accelerometer leveraging silicon based three-electrode structure: (**a**) The simulation model of the mechanical module was composed of the annular flow channel, the electrolyte, and the electrode based sensitive unit, where angular accelerations were used as inputs, and electrolyte velocities around sensitive microelectrodes were obtained as outputs (**b**). (**c**) The simulation model of the electromechanical module included multiple orifices covered by three sensitive microelectrodes filled with the electrolyte, where electrolyte velocities around sensitive microelectrodes obtained in the simulation of the mechanical module were used as inputs, and generated currents of sensitive microelectrodes were obtained as outputs (**d**).

More specifically, the simulation model of the mechanical module is shown in Figure 2a, which was composed of the annular flow channel, the electrolyte, and the electrode-based sensitive unit. The physical fields of rotating machinery and laminar flow were used for coupling, where angular accelerations were used as inputs, and electrolyte velocities around sensitive microelectrodes were obtained as outputs (see Figure 2b).

In the mechanical module, the electrolyte’s material, dynamic viscosity, and density were set to water, 8.28 × 10^−4^ Pa·s and 1410 kg/m^3^, respectively. The initial velocity of the electrolyte and pressure were set to 0 m/s and 0 Pa, respectively. The boundary was set to an open boundary, and the normal stress of the boundary was set to 0 N/m^2^. At the same time, each wall of the model was set as a rotating wall. The angular velocities of the rotating domain were set as a time-varying sine function with an amplitude of 1 × 10^−4^ m/s, through which the input angular accelerations could be obtained.

In addition, the simulation model of the electromechanical module is shown in Figure 2c, which included multiple orifices covered by three sensitive microelectrodes filled with the electrolyte. The physics fields of laminar flow and tertiary current distribution were selected for simulation modeling, where electrolyte velocities around sensitive microelectrodes obtained in the simulation of the mechanical module were used as inputs, and generated currents of sensitive microelectrodes were obtained as outputs (see Figure 2d).

In the electromechanical module, the electrolyte conductivity of the electrolyte was set to 1 × 10^5^ S/m, and the rest of the properties of the electrolyte were the same as those of the mechanical module. In laminar flow physics, the initial velocity of the electrolyte and pressure were consistent with the mechanical module. In the tertiary current distribution physics field, the concentrations of ions c1 and c2 involved in the reaction were set to 4000 mol/m^3^ and 40 mol/m^3^, respectively, corresponding to ions ${\;I}^{-}$ and $I_{3}{}^{-}$. The velocity of the electrolyte at the inlet was set to a time-varying sinusoidal function with an amplitude of 1 × 10^−4^ m/s.

Then, the results of the combined simulation of both the mechanical and the electrochemical modules were used as the basis for the selection of the final key geometrical parameters, where angular accelerations were used as inputs, and generated currents of sensitive microelectrodes were obtained as outputs.

**Figure 3:**
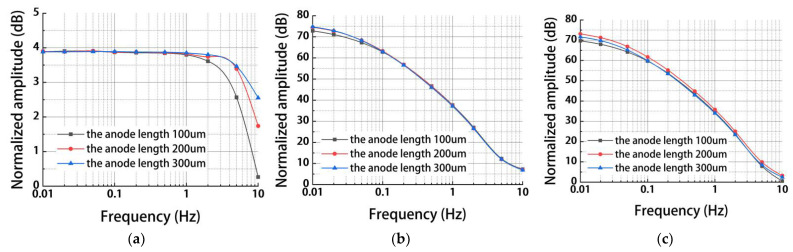
Simulation results of (**a**) the mechanical module, (**b**) the electrochemical module, and (**c**) the coupled modules for the MEMS electrochemical angular accelerometer leveraging silicon-based three-electrode structure as a function of anode length $L_{a}$ (100 μm, 200 μm, 300 μm) under the conditions of orifice diameter $d_{m}$ of 80 μm and orifice spacing $L_{s}$ of 380 μm.

Figure 3 shows simulation results of the MEMS electrochemical angular accelerometer leveraging silicon based three-electrode structure as a function of anode length $L_{a}$ (100 μm, 200 μm, 300 μm) under the conditions of orifice diameter $d_{m}$ of 80 μm and orifice spacing $L_{s}$ of 380 μm. For the mechanical module, in the frequency range of 0.01–5 Hz, the reduction of $L_{a}$ had a very small effect on the outputs of electrolyte velocities, while in the frequency range of 5–10 Hz, increasing the anode length increased electrolyte velocities (see Figure 3a). This is because the increase in $L_{a}$ leads to an increase in flow resistance and corresponding increase in the bandwidth at the high-frequency domain [13].

For the electrochemical module, the influences of $L_{a}$ on the outputs of generated currents of sensitive microelectrodes were negligible (see Figure 3b). As to the results of the combined simulation of both the mechanical and the electrochemical modules, the change in anode length had limited effects (2 dB variation from 100 to 300 μm of $L_{a}$) on the outputs of generated currents of sensitive microelectrodes (see Figure 3c), since the electrochemical module played a much more important role (≈70 dB at 0.01 Hz) than the mechanical module (≈4.5 dB at 0.01 Hz). Since the outputs of generated currents of sensitive microelectrodes with an anode length of 200 um were greater than that of 100 um and 300 um (see Figure 3c), which indicated a higher sensitivity, the anode length was selected as 200 um.

**Figure 4:**
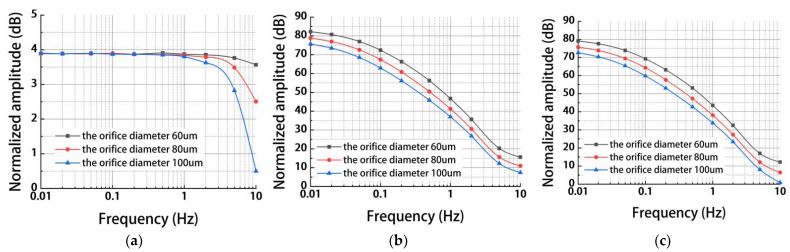
Simulation results of (**a**) the mechanical module, (**b**) the electrochemical module, and (**c**) the coupled modules for the MEMS electrochemical angular accelerometer leveraging silicon-based three-electrode structure as a function of orifice diameter $d_{m}$ (60 μm, 80 μm, 100 μm) under the conditions of anode length $L_{a}$ of 200 μm and orifice spacing $L_{s}$ of 500 μm.

Figure 4 shows simulation results of a MEMS electrochemical angular accelerometer leveraging silicon-based three-electrode structure as a function of orifice diameter $d_{m}$ (60 μm, 80 μm, 100 μm) under the conditions of anode length $L_{a}$ of 200 μm and orifice spacing $L_{s}$ of 500 μm. For the mechanical module, a similar trend was observed between $L_{a}$ and $d_{m}$ (see Figure 4a) because of the same mechanism through which variations in flow resistance can affect the working bandwidth of the angular accelerometer.

For the electrochemical module, the decrease in $d_{m}$ was observed to increase the outputs of generated currents because of enlarged areas of sensitive microelectrodes (see Figure 4b). Again, since the electrochemical module dominated the simulation process, results of the combined simulation of both the mechanical and the electrochemical modules (see Figure 4c) were consistent with what was found in Figure 4b. Therefore, a smaller orifice diameter could increase the outputs of generated currents of sensitive microelectrodes. At the same time, the limitation of microfabrication was taken into consideration, so the orifice diameter was selected as 80 μm.

**Figure 5:**
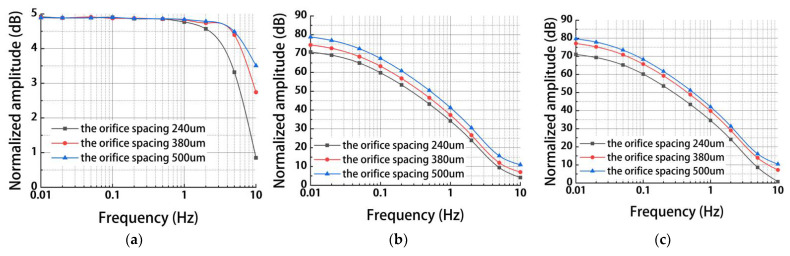
Simulation results of (**a**) the mechanical module, (**b**) the electrochemical module, and (**c**) the coupled modules for the MEMS electrochemical angular accelerometer leveraging silicon-based three-electrode structure as a function of orifice spacing $L_{s}$ (240 μm, 380 μm, 500 μm) under the conditions of anode length $L_{a}$ of 200 μm and orifice diameter $d_{m}$ of 80 μm.

Figure 5 shows simulation results of MEMS electrochemical angular accelerometer leveraging silicon-based three-electrode structure as a function of orifice spacing $L_{s}$ (240 μm, 380 μm, 500 μm) under the conditions of anode length $L_{a}$ of 200 μm and orifice diameter $d_{m}$ of 80 μm. For the mechanical module, a similar trend was observed among $L_{a}$, $d_{m}$, and $L_{s}$ (see Figure 5a) because variations in flow resistance can affect the working bandwidth of the angular accelerometer.

For the electrochemical module coupled with or without the mechanical module, similar with $d_{m}$, the increase in $L_{s}$ was observed to increase the outputs of generated currents because of enlarged areas of sensitive microelectrodes (see Figure 5b,c). In order to validate the results of numerical simulations, $L_{s}$ of 240 μm, 380 μm, and 500 μm were included in the fabrication and characterization of the electrochemical angular micro-accelerometer developed in this study.

## 4. Fabrication and Packaging

**Figure 6:**
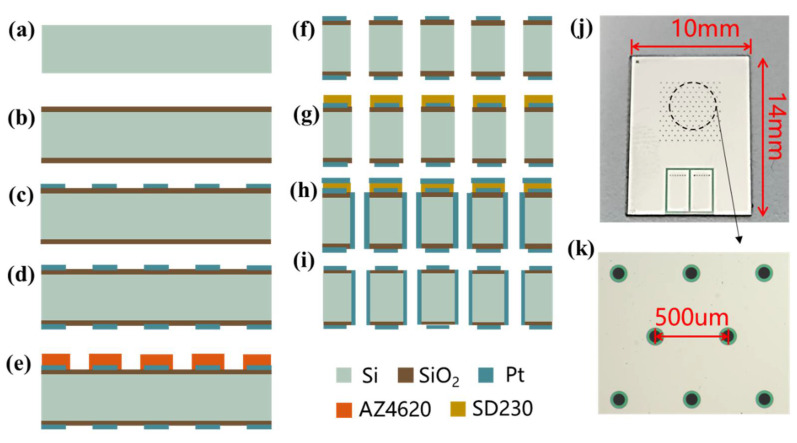
Microfabrication of the MEMS electrochemical angular accelerometer leveraging silicon based three-electrode structure including key steps of wafer cleaning (**a**), thermal oxidation (**b**), lift-off on the front side (**c**), lift-off on the back side (**d**), DRIE (**e**,**f**), sidewall sputtering (**g**–**i**). Pictures of the fabricated sensitive unit (**j**) with enlarged view of flow orifices (**k**).

Figure 6a–i is a MEMS process flow to fabricate the electrochemical angular micro-accelerometer. First the thickness, the size, and the resistivity of the silicon wafer were selected as 200 μm, 4 inches, and 0.0015 Ω·cm. Then, the silicon wafer was thoroughly cleaned by boiled acid and water (a). By means of thermal oxygen, 700 nm thick silicon oxide was grown on the surface of the silicon wafer as an insulating layer (b). After the treatment of oxygen plasma, positive photoresist AZ1500 was applied on the front surface of the silicon wafer for spin-off, pre-baking, exposure, and development. Through the process of electron beam evaporation, 300 Å Ti and 2500 Å Pt were deposited on the front surface of the silicon wafer, which was followed by lift-off (c). Similar operations were performed on the back side to form the same metal patterns on the front and back (d). A positive photoresist AZ4620 was applied on the front side of the silicon wafer for spin-off, pre-baking, exposure with alignment, and development (e). Reactive ion etching was conducted to pattern silicon oxide on the front side, which was followed by deep reactive ion etching to form through-holes in the silicon wafer (f). AZ230 was attached to the front side of the patterned silicon wafer as a film, which was pre-baked, exposed, and developed (g). 300 Å Ti and 2500 Å Pt were sputtered from the front side of the silicon wafer, which was followed by the peel of AZ230 (h,i).

A fabricated sensitive unit is shown in Figure 6j, with a length of 10 mm and a width of 14 mm. An enlarged view focusing on flow orifices of the sensitive unit is shown in Figure 6k, with an orifice spacing of 500 μm, an orifice diameter of 80 μm, and an insulation distance of 20 μm.

**Figure 7:**
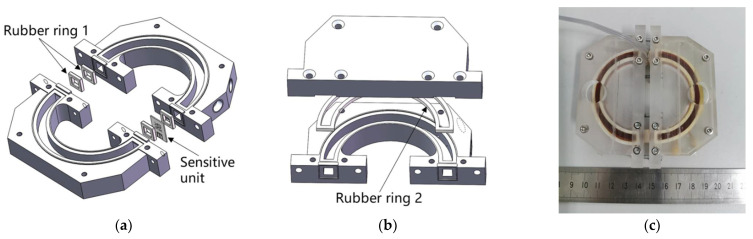
Packaging method of the MEMS electrochemical angular accelerometer leveraging silicon based three-electrode structure: (**a**) The method of fixing the sensitive unit in the angular flow channel, (**b**) The method of fixing the upper and lower parts of the plexiglass casing and the rubber ring 2, (**c**) A prototype of the fabricated MEMS electrochemical angular accelerometer.

Then, the sensitive unit was packaged by mechanical compression. Figure 7a shows the method of fixing the sensitive unit in the angular flow channel. The rubber ring 1 and the sensitive unit were pressed tightly between the two glass casings by screws to form a complete angular flow channel. Then, as shown in Figure 7b, the upper and lower parts of the plexiglass casing and the rubber ring 2 were sealed with screws. Finally, by injecting electrolyte, the air bubbles in the angular flow channel were expelled, and the injection port was sealed with plastic screws. The electrolyte is a mixed solution of $I_{2}$ and KI. A prototype of the fabricated MEMS electrochemical angular accelerometer is shown in Figure 7c.

## 5. Results

The fabricated MEMS electrochemical angular accelerometers leveraging the silicon-based three-electrode structure were characterized by a standard angular acceleration turntable. The amplitude and frequency of inputting angular acceleration were 0.00316 to 7.90 rad/s^2^, 0.01–10 Hz respectively. In self-noise characterization, the MEMS electrochemical angular accelerometers leveraging silicon-based three-electrode structure was placed on a flat laboratory floor and sampled by a data acquisition card at night.

**Figure 8:**
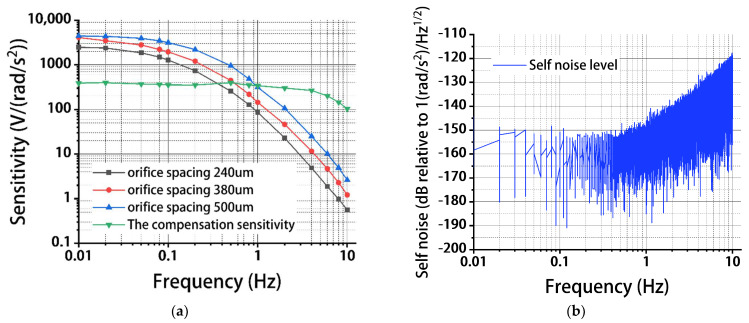
Sensitivities (**a**) and self-noise levels (**b**) of MEMS electrochemical angular accelerometers leveraging a silicon-based three-electrode structure under the conditions of 200 μm for the anode length, 80 μm for the orifice diameter, and 240 μm, 380 μm, and 500 μm for the orifice spacing with and without circuit compensation.

Figure 8a shows sensitivities of the angular accelerometers (with and without circuit compensation) as a function of frequency under the conditions of 200 μm for the anode length, 80 μm for the orifice diameter, and 240 μm, 380 μm, and 500 μm for the orifice spacing. As shown in Figure 8a, the sensitivities of the angular accelerometers without circuit compensation were found to decrease with the increasing frequencies. This is consistent with numerical simulations where both the mechanical and electrochemical modules can be treated as low-pass filters.

In addition, the increase of the orifice spacing was shown to increase the sensitivities of the angular accelerometer. More specifically, the sensitivities were measured as 2468.648 V/(rad/s^2^) vs. 4092.825 V/(rad/s^2^) vs. 4412.429 V/(rad/s^2^) at 0.01 Hz, 1280.578 V/(rad/s^2^) vs. 1956.275 V/(rad/s^2^) vs. 3150.168 V/(rad/s^2^) at 0.1 Hz, 86.443 V/(rad/s^2^) vs. 144.088 V/(rad/s^2^) vs. 314.470 V/(rad/s^2^) at 1 Hz for the orifice spacing of 240 μm vs. 380 μm vs. 500 μm. This is consistent with the numerical simulation that the increase in orifice spacing can enlarge areas of sensitive microelectrodes.

Compared with the test results, the difference between the sensitivity of the orifice spacing of 380 μm and the sensitivity of the orifice spacing of 500 μm during simulation was smaller. This is mainly because the simulation model is a two-dimensional model, so the simulation results can only be used to judge the trend of sensitivity change under different geometrical parameters, which is different from the actual three-dimensional structure. However, this difference has little effect and does not affect the final selection of geometrical parameters.

**Figure A1:**
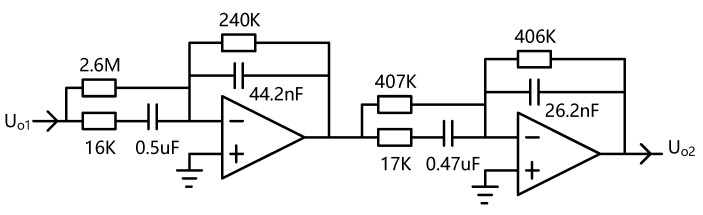
The second-order frequency-compensation circuit of MEMS electrochemical angular accelerometers leveraging a silicon-based three-electrode structure.

Then, circuit compensation was adopted for the angular accelerometer with the orifice spacing of 500 μm, in which the working bandwidth was extended (see Figure A1). More specifically, with a second-order frequency-compensation circuit, the sensitivity and the bandwidth of the device were quantified as 363 V/(rad/s^2^) and 0.01–2 Hz, respectively. This is because the sensitivity of the high frequency was increased by circuit compensation, thereby expanding the bandwidth.

Then, the self-noise level of the angular accelerometer with circuit compensation was tested (see Figure 8b). Within the frequency range of 0.01–2 Hz, a stable self-noise level was found, which was 2.41×10^−8^ (rad/s^2^)/Hz^1/2^ (−152.37 dB) at 0.01 Hz, 1.04×10^−8^ (rad/s^2^)/Hz^1/2^ (−159.62 dB) at 0.1 Hz, and 1.78×10^−8^ (rad/s^2^)/Hz^1/2^ (−154.99 dB) at 1 Hz, because of a stable sensitivity within this frequency domain. Furthermore, when the frequency was increased from 1 to 10 Hz, an increase in the self-noise level was located, resulting from large environmental noises within this frequency domain.

Finally, the performance of the MEMS electrochemical angular accelerometer leveraging silicon-based three-electrode structure developed in this paper was compared with the previously reported electrochemical angular accelerometers [16,17,18] (see Table 1). In comparison to [16], higher sensitivities before and after leveling and a lower self-noise level were quantified as 1 V/(rad/s^2^) vs. 2.405 V/(rad/s^2^) @10 Hz, 8 vs. 363 V/(rad/s^2^), 5.62 × 10^−6^ vs. 1.78 × 10^−8^ (rad/s^2^)/Hz^1/2^ @1 Hz. This is mainly because the sensitive unit of the electrochemical angular accelerometer reported in [16] was fabricated by ceramic sintering, which cannot be effectively optimized by adjusting geometrical dimensions.

In comparison to previously developed MEMS electrochemical angular accelerometers based on planar electrodes [17,18], this study was featured with a higher sensitivity (2.405 V/(rad/s^2^) vs. 0.033 V/(rad/s^2^) vs. 0.051 V/(rad/s^2^) at 10 Hz) and a lower self-noise level (1.78 × 10^−8^ (rad/s^2^)/Hz^1/2^ vs. 1.17 × 10^−6^ (rad/s^2^)/Hz^1/2^ vs. 8.91 × 10^−7^ (rad/s^2^)/Hz^1/2^ at 10 Hz). This is mainly because in this study, the silicon based three-electrode structure was adopted, enabling full contact and reaction between sensitive microelectrodes and the electrolyte solution, leading to a large output current.

## 6. Conclusions

In this study, a MEMS electrochemical angular accelerometer based on silicon conductivity was fabricated. The electrode performance under different orifice spacing and bias voltage was tested, and the consistency of the sensitivity of the optimal parameter electrode was tested. The sensitivity at 0.01 Hz is 4413.053 V/(rad/s^2^), the sensitivity at 1 Hz is 290.193 V/(rad/s^2^), the 3 dB bandwidth is 0.01–2 Hz, and the sensitivity within the working bandwidth is 363 V/(rad/s^2^). At the same time, it has a lower self-noise level of 5.04 × 10^−8^ (rad/s^2^)/Hz^1/2^ @1 Hz, which is higher than the previous devices in sensitivity and resolution, and it has better performance.

## Figures and Tables

**Table 1:** Comparison of key parameters of electrochemical angular accelerometers.

Performance Parameter	Unit	[16]	[17]	[18]	This Device
Original sensitivity	V/(rad/s^2^)	1 @10 Hz	0.033 @10 Hz	0.051 @10 Hz	2.405@10 Hz
Sensitivity after leveling	V/(rad/s^2^)	8	10	22	363
Self-noise level	(rad/s^2^)/Hz^1/2^	5.62 × 10^−6^ @1 Hz	1.17 × 10^−6^ @1 Hz	8.91 × 10^−7^ @1 Hz	1.78 × 10^−8^ @1 Hz

## Appendix A

The differential voltage output $U_{o1}$ is converted to a differential voltage output $U_{o2}$ through a second-order frequency-compensation circuit to expand the bandwidth of the angular accelerometers developed in this paper.
